# Bis{1,2-bis­[2-(1*H*-imidazol-1-yl)eth­oxy]ethane-κ^2^ *N* ^3^,*N* ^3′^}dichloridocadmium(II) monohydrate

**DOI:** 10.1107/S1600536810019148

**Published:** 2010-05-29

**Authors:** Guang-Xiang Liu

**Affiliations:** a Anhui Key Laboratory of Functional Coordination Compounds, School of Chemistry and Chemical Engineering, Anqing Normal University, Anqing 246003, People’s Republic of China

## Abstract

The asymmetric unit of the title compound, [CdCl_2_(C_12_H_18_N_4_O_2_)_2_]·H_2_O, contains one water mol­ecule and two halves of a [CdCl_2_(BIEE)_2_] complex mol­ecule {BIEE is 1,2-bis­[2-(1*H*-imidazol-1-yl)eth­oxy]ethane}, with the Cd^II^ atoms lying on inversion centres. Each metal atom displays an elongated octa­hedral coordination geometry provided by two *trans*-arranged chloride anions and four N atoms from two BIEE ligands. Weak O—H⋯Cl hydrogen-bond inter­actions contribute to the stability of the crystal packing.

## Related literature

For general background to flexible bis­(imidazole) ligands, see: Liu *et al.* (2007 ▶); Wen *et al.* (2007 ▶); Jin *et al.* (2006 ▶). For a related structure, see: Liu *et al.* (2010 ▶). 
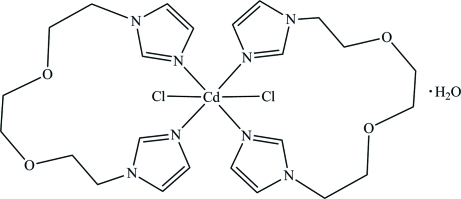


## Experimental

### 

#### Crystal data

[CdCl_2_(C_12_H_18_N_4_O_2_)_2_]·H_2_O
*M* *_r_* = 701.92
Monoclinic, 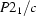

*a* = 15.3629 (13) Å
*b* = 11.0659 (9) Å
*c* = 18.4492 (16) Å
β = 102.558 (1)°
*V* = 3061.4 (4) Å^3^
*Z* = 4
Mo *K*α radiation
μ = 0.94 mm^−1^
*T* = 293 K
0.26 × 0.22 × 0.20 mm

#### Data collection

Bruker SMART APEX CCD area-detector diffractometer
Absorption correction: multi-scan (*SADABS*; Bruker, 2000 ▶) *T*_min_ = 0.793, *T*_max_ = 0.835
21862 measured reflections
5691 independent reflections
4148 reflections with *I* > 2σ(*I*)
*R*_int_ = 0.022

#### Refinement

*R*[*F* ^2^ > 2σ(*F* ^2^)] = 0.030
*wR*(*F* ^2^) = 0.087
*S* = 1.04
5691 reflections
372 parameters
8 restraints
H atoms treated by a mixture of independent and constrained refinement
Δρ_max_ = 0.53 e Å^−3^
Δρ_min_ = −0.38 e Å^−3^

### 

Data collection: *SMART* (Bruker, 2000 ▶); cell refinement: *SAINT* (Bruker, 2000 ▶); data reduction: *SAINT*; program(s) used to solve structure: *SHELXS97* (Sheldrick, 2008 ▶); program(s) used to refine structure: *SHELXL97* (Sheldrick, 2008 ▶); molecular graphics: *SHELXTL* (Sheldrick, 2008 ▶); software used to prepare material for publication: *SHELXTL*.

## Supplementary Material

Crystal structure: contains datablocks I, global. DOI: 10.1107/S1600536810019148/rz2451sup1.cif

Structure factors: contains datablocks I. DOI: 10.1107/S1600536810019148/rz2451Isup2.hkl

Additional supplementary materials:  crystallographic information; 3D view; checkCIF report

## Figures and Tables

**Table 1:** Hydrogen-bond geometry (Å, °)

*D*—H⋯*A*	*D*—H	H⋯*A*	*D*⋯*A*	*D*—H⋯*A*
O1*W*—H1*WB*⋯Cl2^i^	0.85 (2)	2.33 (3)	3.165 (3)	164 (5)
O1*W*—H1*WA*⋯Cl1^ii^	0.88 (6)	2.42 (5)	3.198 (4)	147 (7)

Symmetry codes: (i) 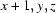; (ii) 
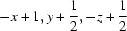
.

**Fig. 1.**
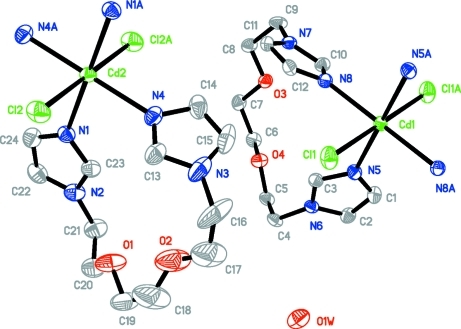
The asymmetric unit of the title compound, with the atom-numbering scheme. Displacement ellipsoids are drawn at the 30% probability level. Hydrogen atoms are omitted for clarity.

**Fig. 2.**
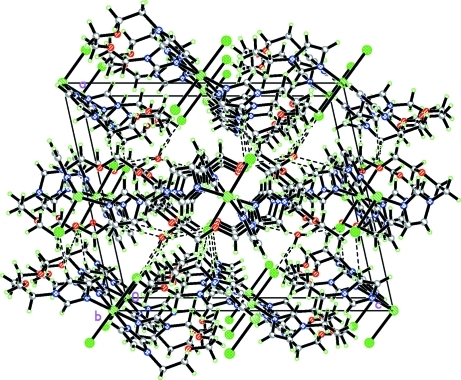
Packing diagram of the title compound viewed along the b axis. Hydrogen bonds are shown as dashed lines.

## supplementary crystallographic information

### Comment

A large number of beautiful metal organic frameworks (MOFs) of ingenious design based on flexible bis(imidazole) ligands, such as (N-im)_2_(CH_2_)_n_ (n = 1-4), have recently been constructed (Liu *et al.*, 2007; Wen *et al.*, 2007; Jin *et al.*, 2006). These ligands bearing alkyl spacers are good choices of N-donor ligands, because the flexible nature of the spacers allows the ligands to bend and rotate when it coordinates to metal centers. The structures and properties also can be modified by changing the spacer groups, for an instance, by varying the length of the spacer. We designed and prepared a long ligand, 1,2-bis(2-(1*H*-imidazol-1-yl)ethoxy)ethane (BIEE), which is longer than 1,1'-(2,2'-oxybis(ethane-2,1-diyl))bis(1*H*-imidazole)) (obbm). The increasing length may control the physical dimensions of the crystalline architecture and, accordingly, affects the internal chemistry of the coordination polymers. Therefore, the exploration of this ligand is necessary in order to enrich and develop this field.

The molecular structure of the title compound is shown in Fig. 1. The asymmetric unit contains one water molecule and two crystallographically independent half of a [CdCl_2_(BIEE)_2_] complex molecule, with the metal atoms lying on inversion centres. Each cadmium(II) atom displays an elongated octahedral coordination geometry, with four N atoms from two BIEE ligands providing the equatorial plane and two Cl anions at the axial positions. The Cd—N lengths range from 2.328 (2) to 2.365 (2) Å; these values agree well with those observed in [Cd(NCS)_2_(1-vinylimidazole)_4_] (Liu *et al.*, 2010). The values of the bond angles around the cadmium atoms are close to those expected for a regular octahedral geometry, the largest deviation being observed for the N8—Cd1—N5 angle [91.68 (8)°]. Weak O—H···Cl interactions (Table 1) contribute to the stability of the crystal packing (Fig. 2).

### Experimental

An aqueous solution (15 ml) of CdCl_2_.2.5H_2_O (0.23 g, 1.0 mmol) was added slowly with constant stirring to a solution of 1,1'-(2,2'-oxybis(ethane-2,1-diyl))bis(1*H*-imidazole)) (0.21 g, 0.1 mmol) in water (20 ml). The reaction mixture was then heated to reflux for 3 h. The resulting mixture was left to stand at room temperature for three weeks. Colourless block crystals suitable for X-ray analysis were obtained on slow evaporation of the solvent. Yield: 67% (based on Cd).

### Refinement

The water H atoms were located in a difference Fourier map and refined with the O—H bond distances restrained to 0.86 Å. All other H atoms were positioned geometrically, with C—H = 0.93–0.97 Å, and constrained to ride on their parent atoms, with U_iso_(H) = 1.2 U_eq_(C).

### Crystal data

**Table:**

[CdCl_2_(C_12_H_18_N_4_O_2_)_2_]·H_2_O	*F*(000) = 1440
*M**_r_* = 701.92	*D*_x_ = 1.523 Mg m^−^^3^
Monoclinic, *P*2_1_/*c*	Mo *K*α radiation, λ = 0.71073 Å
Hall symbol: -P 2ybc	Cell parameters from 9598 reflections
*a* = 15.3629 (13) Å	θ = 2.2–27.2°
*b* = 11.0659 (9) Å	µ = 0.94 mm^−^^1^
*c* = 18.4492 (16) Å	*T* = 293 K
β = 102.558 (1)°	Block, colorless
*V* = 3061.4 (4) Å^3^	0.26 × 0.22 × 0.20 mm
*Z* = 4	

### Data collection

**Table:**

Bruker SMART APEX CCD area-detector diffractometer	5691 independent reflections
Radiation source: sealed tube	4148 reflections with *I* > 2σ(*I*)
graphite	*R*_int_ = 0.022
phi and ω scans	θ_max_ = 25.5°, θ_min_ = 1.4°
Absorption correction: multi-scan (*SADABS*; Bruker, 2000)	*h* = −18→18
*T*_min_ = 0.793, *T*_max_ = 0.835	*k* = −13→13
21862 measured reflections	*l* = −22→22

### Refinement

**Table:**

Refinement on *F*^2^	Primary atom site location: structure-invariant direct methods
Least-squares matrix: full	Secondary atom site location: difference Fourier map
*R*[*F*^2^ > 2σ(*F*^2^)] = 0.030	Hydrogen site location: inferred from neighbouring sites
*wR*(*F*^2^) = 0.087	H atoms treated by a mixture of independent and constrained refinement
*S* = 1.04	*w* = 1/[σ^2^(*F*_o_^2^) + (0.0331*P*)^2^ + 3.6911*P*] where *P* = (*F*_o_^2^ + 2*F*_c_^2^)/3
5691 reflections	(Δ/σ)_max_ < 0.001
372 parameters	Δρ_max_ = 0.53 e Å^−^^3^
8 restraints	Δρ_min_ = −0.38 e Å^−^^3^

### Special details

**Table:**

Geometry. All esds (except the esd in the dihedral angle between two l.s. planes) are estimated using the full covariance matrix. The cell esds are taken into account individually in the estimation of esds in distances, angles and torsion angles; correlations between esds in cell parameters are only used when they are defined by crystal symmetry. An approximate (isotropic) treatment of cell esds is used for estimating esds involving l.s. planes.
Refinement. Refinement of *F*^2^ against ALL reflections. The weighted *R*-factor wR and goodness of fit *S* are based on *F*^2^, conventional *R*-factors *R* are based on *F*, with *F* set to zero for negative *F*^2^. The threshold expression of *F*^2^ > σ(*F*^2^) is used only for calculating *R*-factors(gt) etc. and is not relevant to the choice of reflections for refinement. *R*-factors based on *F*^2^ are statistically about twice as large as those based on *F*, and *R*- factors based on ALL data will be even larger.

### Fractional atomic coordinates and isotropic or equivalent isotropic displacement parameters (Å^2^)

**Table:**

	*x*	*y*	*z*	*U*_iso_*/*U*_eq_	
Cd1	0.5000	0.0000	0.5000	0.03408 (9)	
Cd2	0.0000	0.5000	0.5000	0.03731 (10)	
Cl1	0.35374 (6)	0.07994 (8)	0.40345 (5)	0.0623 (3)	
Cl2	−0.14291 (6)	0.51856 (10)	0.38908 (5)	0.0654 (3)	
N1	0.06622 (17)	0.6671 (2)	0.45294 (15)	0.0453 (6)	
N2	0.1595 (2)	0.7621 (3)	0.39710 (16)	0.0555 (7)	
N3	0.0872 (3)	0.2692 (3)	0.3298 (2)	0.0799 (11)	
N4	0.06263 (18)	0.3669 (3)	0.42699 (17)	0.0508 (7)	
N5	0.58186 (17)	0.1608 (2)	0.46622 (14)	0.0398 (6)	
N6	0.61728 (16)	0.3470 (2)	0.44305 (13)	0.0373 (6)	
N7	0.48013 (18)	0.2327 (2)	0.69235 (13)	0.0394 (6)	
N8	0.46279 (17)	0.1245 (2)	0.59039 (13)	0.0386 (6)	
O1	0.1550 (2)	0.6593 (3)	0.25676 (15)	0.0883 (10)	
O2	0.1213 (4)	0.4214 (4)	0.2164 (2)	0.148 (2)	
O3	0.60598 (15)	0.42888 (19)	0.70446 (12)	0.0457 (5)	
O4	0.62769 (15)	0.51267 (18)	0.56526 (12)	0.0456 (5)	
O1W	0.6777 (2)	0.5003 (4)	0.26691 (19)	0.0850 (10)	
C1	0.6641 (2)	0.1620 (3)	0.44932 (19)	0.0488 (8)	
H1	0.6993	0.0940	0.4476	0.059*	
C2	0.6870 (2)	0.2762 (3)	0.43540 (18)	0.0460 (8)	
H2	0.7398	0.3012	0.4231	0.055*	
C3	0.5561 (2)	0.2744 (3)	0.46206 (16)	0.0392 (7)	
H3	0.5021	0.3010	0.4712	0.047*	
C4	0.6131 (2)	0.4790 (3)	0.43723 (17)	0.0444 (8)	
H4A	0.5515	0.5049	0.4304	0.053*	
H4B	0.6348	0.5043	0.3940	0.053*	
C5	0.6676 (2)	0.5382 (3)	0.50518 (17)	0.0436 (7)	
H5A	0.7281	0.5073	0.5152	0.052*	
H5B	0.6697	0.6248	0.4977	0.052*	
C6	0.6773 (2)	0.5605 (3)	0.63323 (17)	0.0454 (8)	
H6A	0.6921	0.6444	0.6267	0.055*	
H6B	0.7324	0.5156	0.6491	0.055*	
C7	0.6227 (2)	0.5509 (3)	0.68994 (18)	0.0472 (8)	
H7A	0.6538	0.5895	0.7354	0.057*	
H7B	0.5666	0.5927	0.6726	0.057*	
C8	0.5455 (2)	0.4203 (3)	0.75265 (17)	0.0462 (8)	
H8A	0.4913	0.4643	0.7318	0.055*	
H8B	0.5720	0.4555	0.8005	0.055*	
C9	0.5243 (2)	0.2903 (3)	0.76207 (16)	0.0499 (8)	
H9A	0.5791	0.2472	0.7826	0.060*	
H9B	0.4860	0.2841	0.7974	0.060*	
C10	0.5194 (2)	0.1621 (3)	0.65024 (16)	0.0408 (7)	
H10	0.5796	0.1418	0.6618	0.049*	
C11	0.3925 (2)	0.2424 (3)	0.65710 (17)	0.0442 (8)	
H11	0.3485	0.2863	0.6729	0.053*	
C12	0.3827 (2)	0.1752 (3)	0.59443 (17)	0.0416 (7)	
H12	0.3296	0.1651	0.5595	0.050*	
C13	0.0398 (3)	0.3551 (4)	0.3545 (2)	0.0640 (10)	
H13	−0.0041	0.4010	0.3240	0.077*	
C14	0.1286 (2)	0.2831 (3)	0.4502 (3)	0.0665 (11)	
H14	0.1580	0.2696	0.4991	0.080*	
C15	0.1439 (3)	0.2235 (4)	0.3904 (4)	0.0868 (16)	
H15	0.1856	0.1624	0.3906	0.104*	
C16	0.0786 (4)	0.2352 (6)	0.2505 (3)	0.131 (3)	
H16A	0.1019	0.1540	0.2490	0.157*	
H16B	0.0156	0.2324	0.2273	0.157*	
C17	0.1193 (6)	0.3070 (6)	0.2090 (3)	0.139 (3)	
H17A	0.1807	0.2800	0.2169	0.167*	
H17B	0.0920	0.2903	0.1575	0.167*	
C18	0.1213 (7)	0.5041 (6)	0.1698 (3)	0.153 (3)	
H18A	0.0604	0.5098	0.1416	0.184*	
H18B	0.1561	0.4735	0.1358	0.184*	
C19	0.1489 (4)	0.6200 (5)	0.1849 (3)	0.0984 (16)	
H19A	0.1081	0.6730	0.1520	0.118*	
H19B	0.2071	0.6290	0.1730	0.118*	
C20	0.1985 (3)	0.7675 (4)	0.2759 (2)	0.0749 (12)	
H20A	0.1580	0.8338	0.2585	0.090*	
H20B	0.2485	0.7738	0.2518	0.090*	
C21	0.2309 (3)	0.7774 (4)	0.3559 (2)	0.0711 (11)	
H21A	0.2583	0.8561	0.3675	0.085*	
H21B	0.2763	0.7166	0.3723	0.085*	
C22	0.1212 (3)	0.8467 (3)	0.4339 (2)	0.0635 (10)	
H22	0.1321	0.9295	0.4352	0.076*	
C23	0.1232 (2)	0.6563 (3)	0.40966 (19)	0.0506 (8)	
H23	0.1369	0.5832	0.3899	0.061*	
C24	0.0646 (2)	0.7880 (3)	0.4682 (2)	0.0588 (9)	
H24	0.0299	0.8241	0.4976	0.071*	
H1WA	0.678 (5)	0.550 (6)	0.230 (3)	0.19 (3)*	
H1WB	0.7311 (18)	0.505 (5)	0.292 (3)	0.12 (2)*	

### Atomic displacement parameters (Å^2^)

**Table:**

	*U*^11^	*U*^22^	*U*^33^	*U*^12^	*U*^13^	*U*^23^
Cd1	0.04100 (18)	0.02596 (15)	0.03493 (17)	−0.00286 (12)	0.00747 (13)	−0.00432 (12)
Cd2	0.03589 (17)	0.04115 (18)	0.03550 (17)	−0.00300 (13)	0.00907 (13)	0.00258 (13)
Cl1	0.0565 (5)	0.0578 (5)	0.0629 (5)	0.0116 (4)	−0.0079 (4)	−0.0091 (4)
Cl2	0.0485 (5)	0.0866 (7)	0.0535 (5)	−0.0051 (5)	−0.0056 (4)	0.0107 (5)
N1	0.0415 (15)	0.0459 (15)	0.0483 (15)	−0.0039 (12)	0.0091 (12)	0.0062 (13)
N2	0.0594 (19)	0.0520 (17)	0.0558 (18)	−0.0209 (15)	0.0145 (15)	0.0010 (14)
N3	0.091 (3)	0.061 (2)	0.107 (3)	−0.018 (2)	0.062 (2)	−0.030 (2)
N4	0.0454 (16)	0.0502 (16)	0.0607 (18)	−0.0017 (13)	0.0199 (14)	−0.0053 (14)
N5	0.0420 (14)	0.0334 (13)	0.0442 (14)	−0.0046 (11)	0.0096 (12)	−0.0012 (11)
N6	0.0453 (15)	0.0339 (13)	0.0328 (13)	−0.0049 (11)	0.0086 (11)	0.0011 (10)
N7	0.0552 (17)	0.0327 (13)	0.0325 (13)	−0.0055 (12)	0.0142 (12)	−0.0016 (10)
N8	0.0483 (15)	0.0322 (13)	0.0361 (13)	−0.0023 (11)	0.0112 (12)	−0.0025 (11)
O1	0.111 (2)	0.103 (2)	0.0575 (17)	−0.053 (2)	0.0321 (16)	−0.0108 (16)
O2	0.293 (6)	0.086 (3)	0.085 (3)	−0.037 (3)	0.083 (3)	−0.021 (2)
O3	0.0581 (14)	0.0379 (12)	0.0451 (12)	−0.0010 (10)	0.0197 (11)	−0.0046 (10)
O4	0.0514 (13)	0.0461 (13)	0.0399 (12)	−0.0151 (10)	0.0112 (10)	−0.0070 (10)
O1W	0.0576 (19)	0.133 (3)	0.0605 (19)	0.0083 (19)	0.0039 (16)	−0.016 (2)
C1	0.0482 (19)	0.0413 (18)	0.058 (2)	0.0046 (15)	0.0131 (16)	−0.0043 (16)
C2	0.0413 (18)	0.0467 (18)	0.053 (2)	−0.0050 (15)	0.0175 (15)	−0.0033 (15)
C3	0.0403 (17)	0.0369 (16)	0.0419 (17)	−0.0026 (13)	0.0121 (14)	0.0008 (13)
C4	0.059 (2)	0.0336 (16)	0.0378 (17)	−0.0056 (14)	0.0052 (15)	0.0076 (13)
C5	0.054 (2)	0.0332 (15)	0.0440 (18)	−0.0085 (14)	0.0118 (15)	0.0051 (14)
C6	0.0509 (19)	0.0383 (17)	0.0449 (18)	−0.0109 (15)	0.0057 (15)	−0.0062 (14)
C7	0.054 (2)	0.0383 (17)	0.0477 (19)	−0.0041 (15)	0.0081 (16)	−0.0113 (15)
C8	0.061 (2)	0.0445 (18)	0.0348 (16)	−0.0075 (16)	0.0136 (15)	−0.0097 (14)
C9	0.073 (2)	0.0488 (19)	0.0286 (16)	−0.0135 (17)	0.0135 (15)	−0.0028 (14)
C10	0.0435 (17)	0.0406 (17)	0.0397 (17)	−0.0043 (14)	0.0117 (14)	−0.0021 (14)
C11	0.060 (2)	0.0324 (16)	0.0443 (18)	0.0069 (15)	0.0204 (16)	−0.0002 (14)
C12	0.0473 (18)	0.0330 (16)	0.0422 (17)	0.0031 (14)	0.0049 (14)	0.0038 (13)
C13	0.069 (3)	0.059 (2)	0.068 (3)	−0.004 (2)	0.026 (2)	−0.015 (2)
C14	0.046 (2)	0.053 (2)	0.102 (3)	0.0028 (18)	0.021 (2)	0.006 (2)
C15	0.066 (3)	0.049 (2)	0.162 (5)	−0.003 (2)	0.061 (3)	−0.020 (3)
C16	0.169 (6)	0.119 (5)	0.140 (5)	−0.065 (4)	0.110 (5)	−0.085 (4)
C17	0.235 (8)	0.112 (5)	0.068 (4)	−0.037 (5)	0.028 (4)	−0.029 (3)
C18	0.268 (10)	0.119 (6)	0.057 (3)	−0.041 (6)	0.003 (5)	−0.014 (3)
C19	0.138 (5)	0.107 (4)	0.060 (3)	−0.013 (4)	0.042 (3)	0.001 (3)
C20	0.074 (3)	0.077 (3)	0.080 (3)	−0.019 (2)	0.031 (2)	0.010 (2)
C21	0.066 (3)	0.073 (3)	0.080 (3)	−0.026 (2)	0.028 (2)	0.000 (2)
C22	0.069 (3)	0.043 (2)	0.075 (3)	−0.0130 (19)	0.008 (2)	0.0078 (19)
C23	0.050 (2)	0.0487 (19)	0.055 (2)	−0.0121 (16)	0.0150 (17)	0.0019 (16)
C24	0.058 (2)	0.051 (2)	0.069 (2)	−0.0039 (18)	0.0163 (19)	−0.0040 (18)

### Geometric parameters (Å, °)

**Table:**

Cd1—N8	2.328 (2)	C2—H2	0.9300
Cd1—N8^i^	2.328 (2)	C3—H3	0.9300
Cd1—N5	2.340 (2)	C4—C5	1.499 (4)
Cd1—N5^i^	2.340 (2)	C4—H4A	0.9700
Cd1—Cl1	2.6951 (9)	C4—H4B	0.9700
Cd1—Cl1^i^	2.6952 (9)	C5—H5A	0.9700
Cd2—N4^ii^	2.339 (3)	C5—H5B	0.9700
Cd2—N4	2.339 (3)	C6—C7	1.480 (4)
Cd2—N1	2.365 (3)	C6—H6A	0.9700
Cd2—N1^ii^	2.365 (3)	C6—H6B	0.9700
Cd2—Cl2	2.6639 (9)	C7—H7A	0.9700
Cd2—Cl2^ii^	2.6639 (9)	C7—H7B	0.9700
N1—C23	1.313 (4)	C8—C9	1.493 (4)
N1—C24	1.368 (4)	C8—H8A	0.9700
N2—C23	1.338 (4)	C8—H8B	0.9700
N2—C22	1.363 (5)	C9—H9A	0.9700
N2—C21	1.473 (4)	C9—H9B	0.9700
N3—C13	1.337 (5)	C10—H10	0.9300
N3—C15	1.357 (6)	C11—C12	1.355 (4)
N3—C16	1.487 (6)	C11—H11	0.9300
N4—C13	1.313 (5)	C12—H12	0.9300
N4—C14	1.372 (5)	C13—H13	0.9300
N5—C3	1.315 (4)	C14—C15	1.350 (6)
N5—C1	1.366 (4)	C14—H14	0.9300
N6—C3	1.340 (4)	C15—H15	0.9300
N6—C2	1.360 (4)	C16—C17	1.348 (7)
N6—C4	1.464 (4)	C16—H16A	0.9700
N7—C10	1.335 (4)	C16—H16B	0.9700
N7—C11	1.366 (4)	C17—H17A	0.9700
N7—C9	1.464 (4)	C17—H17B	0.9700
N8—C10	1.316 (4)	C18—C19	1.360 (7)
N8—C12	1.370 (4)	C18—H18A	0.9700
O1—C19	1.378 (5)	C18—H18B	0.9700
O1—C20	1.379 (5)	C19—H19A	0.9700
O2—C18	1.256 (7)	C19—H19B	0.9700
O2—C17	1.273 (7)	C20—C21	1.456 (6)
O3—C7	1.411 (4)	C20—H20A	0.9700
O3—C8	1.422 (4)	C20—H20B	0.9700
O4—C5	1.407 (4)	C21—H21A	0.9700
O4—C6	1.420 (4)	C21—H21B	0.9700
O1W—H1WA	0.88 (6)	C22—C24	1.348 (5)
O1W—H1WB	0.85 (2)	C22—H22	0.9300
C1—C2	1.351 (4)	C23—H23	0.9300
C1—H1	0.9300	C24—H24	0.9300
			
N8—Cd1—N8^i^	180.00 (9)	C7—C6—H6B	110.0
N8—Cd1—N5	88.32 (8)	H6A—C6—H6B	108.3
N8^i^—Cd1—N5	91.68 (8)	O3—C7—C6	110.9 (3)
N8—Cd1—N5^i^	91.68 (8)	O3—C7—H7A	109.5
N8^i^—Cd1—N5^i^	88.32 (8)	C6—C7—H7A	109.5
N5—Cd1—N5^i^	180.0	O3—C7—H7B	109.5
N8—Cd1—Cl1	88.85 (7)	C6—C7—H7B	109.5
N8^i^—Cd1—Cl1	91.15 (7)	H7A—C7—H7B	108.0
N5—Cd1—Cl1	89.63 (7)	O3—C8—C9	109.0 (3)
N5^i^—Cd1—Cl1	90.37 (7)	O3—C8—H8A	109.9
N8—Cd1—Cl1^i^	91.15 (7)	C9—C8—H8A	109.9
N8^i^—Cd1—Cl1^i^	88.85 (7)	O3—C8—H8B	109.9
N5—Cd1—Cl1^i^	90.37 (7)	C9—C8—H8B	109.9
N5^i^—Cd1—Cl1^i^	89.63 (7)	H8A—C8—H8B	108.3
Cl1—Cd1—Cl1^i^	180.0	N7—C9—C8	112.9 (2)
N4^ii^—Cd2—N4	179.999 (1)	N7—C9—H9A	109.0
N4^ii^—Cd2—N1	89.01 (10)	C8—C9—H9A	109.0
N4—Cd2—N1	90.99 (10)	N7—C9—H9B	109.0
N4^ii^—Cd2—N1^ii^	90.99 (10)	C8—C9—H9B	109.0
N4—Cd2—N1^ii^	89.01 (10)	H9A—C9—H9B	107.8
N1—Cd2—N1^ii^	180.0	N8—C10—N7	111.9 (3)
N4^ii^—Cd2—Cl2	91.17 (8)	N8—C10—H10	124.1
N4—Cd2—Cl2	88.84 (8)	N7—C10—H10	124.1
N1—Cd2—Cl2	90.36 (7)	C12—C11—N7	106.2 (3)
N1^ii^—Cd2—Cl2	89.65 (7)	C12—C11—H11	126.9
N4^ii^—Cd2—Cl2^ii^	88.83 (8)	N7—C11—H11	126.9
N4—Cd2—Cl2^ii^	91.17 (8)	C11—C12—N8	109.7 (3)
N1—Cd2—Cl2^ii^	89.64 (7)	C11—C12—H12	125.2
N1^ii^—Cd2—Cl2^ii^	90.35 (7)	N8—C12—H12	125.2
Cl2—Cd2—Cl2^ii^	179.999 (1)	N4—C13—N3	112.0 (4)
C23—N1—C24	105.0 (3)	N4—C13—H13	124.0
C23—N1—Cd2	123.3 (2)	N3—C13—H13	124.0
C24—N1—Cd2	131.4 (2)	C15—C14—N4	108.9 (4)
C23—N2—C22	105.9 (3)	C15—C14—H14	125.5
C23—N2—C21	125.1 (3)	N4—C14—H14	125.5
C22—N2—C21	128.9 (3)	C14—C15—N3	107.2 (4)
C13—N3—C15	106.5 (4)	C14—C15—H15	126.4
C13—N3—C16	125.2 (5)	N3—C15—H15	126.4
C15—N3—C16	128.3 (5)	C17—C16—N3	117.2 (5)
C13—N4—C14	105.4 (3)	C17—C16—H16A	108.0
C13—N4—Cd2	126.7 (3)	N3—C16—H16A	108.0
C14—N4—Cd2	127.9 (3)	C17—C16—H16B	108.0
C3—N5—C1	105.0 (3)	N3—C16—H16B	108.0
C3—N5—Cd1	124.8 (2)	H16A—C16—H16B	107.3
C1—N5—Cd1	130.2 (2)	O2—C17—C16	121.9 (6)
C3—N6—C2	107.1 (3)	O2—C17—H17A	106.9
C3—N6—C4	126.5 (3)	C16—C17—H17A	106.9
C2—N6—C4	126.2 (3)	O2—C17—H17B	106.9
C10—N7—C11	107.0 (2)	C16—C17—H17B	106.9
C10—N7—C9	125.8 (3)	H17A—C17—H17B	106.7
C11—N7—C9	127.2 (3)	O2—C18—C19	126.4 (5)
C10—N8—C12	105.2 (2)	O2—C18—H18A	105.7
C10—N8—Cd1	124.6 (2)	C19—C18—H18A	105.7
C12—N8—Cd1	130.2 (2)	O2—C18—H18B	105.7
C19—O1—C20	116.7 (3)	C19—C18—H18B	105.7
C18—O2—C17	130.9 (5)	H18A—C18—H18B	106.2
C7—O3—C8	110.7 (2)	C18—C19—O1	116.6 (5)
C5—O4—C6	112.2 (2)	C18—C19—H19A	108.1
H1WA—O1W—H1WB	102 (6)	O1—C19—H19A	108.1
C2—C1—N5	110.1 (3)	C18—C19—H19B	108.1
C2—C1—H1	124.9	O1—C19—H19B	108.1
N5—C1—H1	124.9	H19A—C19—H19B	107.3
C1—C2—N6	106.0 (3)	O1—C20—C21	111.4 (3)
C1—C2—H2	127.0	O1—C20—H20A	109.3
N6—C2—H2	127.0	C21—C20—H20A	109.3
N5—C3—N6	111.7 (3)	O1—C20—H20B	109.3
N5—C3—H3	124.1	C21—C20—H20B	109.3
N6—C3—H3	124.1	H20A—C20—H20B	108.0
N6—C4—C5	111.5 (3)	C20—C21—N2	112.7 (3)
N6—C4—H4A	109.3	C20—C21—H21A	109.1
C5—C4—H4A	109.3	N2—C21—H21A	109.1
N6—C4—H4B	109.3	C20—C21—H21B	109.1
C5—C4—H4B	109.3	N2—C21—H21B	109.1
H4A—C4—H4B	108.0	H21A—C21—H21B	107.8
O4—C5—C4	108.1 (2)	C24—C22—N2	107.1 (3)
O4—C5—H5A	110.1	C24—C22—H22	126.4
C4—C5—H5A	110.1	N2—C22—H22	126.4
O4—C5—H5B	110.1	N1—C23—N2	112.5 (3)
C4—C5—H5B	110.1	N1—C23—H23	123.7
H5A—C5—H5B	108.4	N2—C23—H23	123.7
O4—C6—C7	108.7 (3)	C22—C24—N1	109.4 (3)
O4—C6—H6A	110.0	C22—C24—H24	125.3
C7—C6—H6A	110.0	N1—C24—H24	125.3
O4—C6—H6B	110.0		
			
N4^ii^—Cd2—N1—C23	173.1 (3)	C5—O4—C6—C7	169.1 (3)
N4—Cd2—N1—C23	−6.9 (3)	C8—O3—C7—C6	−173.5 (3)
Cl2—Cd2—N1—C23	−95.8 (3)	O4—C6—C7—O3	64.0 (3)
Cl2^ii^—Cd2—N1—C23	84.2 (3)	C7—O3—C8—C9	175.8 (3)
N4^ii^—Cd2—N1—C24	1.1 (3)	C10—N7—C9—C8	100.1 (4)
N4—Cd2—N1—C24	−178.9 (3)	C11—N7—C9—C8	−78.3 (4)
Cl2—Cd2—N1—C24	92.3 (3)	O3—C8—C9—N7	−62.2 (4)
Cl2^ii^—Cd2—N1—C24	−87.7 (3)	C12—N8—C10—N7	0.5 (3)
N1—Cd2—N4—C13	−70.7 (3)	Cd1—N8—C10—N7	179.79 (18)
N1^ii^—Cd2—N4—C13	109.3 (3)	C11—N7—C10—N8	−0.6 (3)
Cl2—Cd2—N4—C13	19.7 (3)	C9—N7—C10—N8	−179.3 (3)
Cl2^ii^—Cd2—N4—C13	−160.3 (3)	C10—N7—C11—C12	0.4 (3)
N1—Cd2—N4—C14	111.2 (3)	C9—N7—C11—C12	179.1 (3)
N1^ii^—Cd2—N4—C14	−68.8 (3)	N7—C11—C12—N8	−0.1 (3)
Cl2—Cd2—N4—C14	−158.5 (3)	C10—N8—C12—C11	−0.2 (3)
Cl2^ii^—Cd2—N4—C14	21.5 (3)	Cd1—N8—C12—C11	−179.4 (2)
N8—Cd1—N5—C3	−40.5 (3)	C14—N4—C13—N3	−0.2 (4)
N8^i^—Cd1—N5—C3	139.5 (3)	Cd2—N4—C13—N3	−178.7 (2)
Cl1—Cd1—N5—C3	48.4 (2)	C15—N3—C13—N4	−0.1 (5)
Cl1^i^—Cd1—N5—C3	−131.6 (2)	C16—N3—C13—N4	−178.9 (4)
N8—Cd1—N5—C1	137.2 (3)	C13—N4—C14—C15	0.4 (4)
N8^i^—Cd1—N5—C1	−42.8 (3)	Cd2—N4—C14—C15	178.8 (2)
Cl1—Cd1—N5—C1	−133.9 (3)	N4—C14—C15—N3	−0.4 (5)
Cl1^i^—Cd1—N5—C1	46.1 (3)	C13—N3—C15—C14	0.3 (5)
N5—Cd1—N8—C10	−68.9 (2)	C16—N3—C15—C14	179.1 (4)
N5^i^—Cd1—N8—C10	111.1 (2)	C13—N3—C16—C17	79.4 (8)
Cl1—Cd1—N8—C10	−158.6 (2)	C15—N3—C16—C17	−99.2 (8)
Cl1^i^—Cd1—N8—C10	21.4 (2)	C18—O2—C17—C16	−147.6 (9)
N5—Cd1—N8—C12	110.1 (2)	N3—C16—C17—O2	−38.2 (12)
N5^i^—Cd1—N8—C12	−69.9 (2)	C17—O2—C18—C19	−159.6 (9)
Cl1—Cd1—N8—C12	20.5 (2)	O2—C18—C19—O1	−18.3 (14)
Cl1^i^—Cd1—N8—C12	−159.5 (2)	C20—O1—C19—C18	168.1 (6)
C3—N5—C1—C2	0.2 (4)	C19—O1—C20—C21	−158.1 (4)
Cd1—N5—C1—C2	−177.9 (2)	O1—C20—C21—N2	−55.9 (5)
N5—C1—C2—N6	−0.6 (4)	C23—N2—C21—C20	76.6 (5)
C3—N6—C2—C1	0.8 (4)	C22—N2—C21—C20	−108.3 (5)
C4—N6—C2—C1	176.5 (3)	C23—N2—C22—C24	1.0 (4)
C1—N5—C3—N6	0.4 (3)	C21—N2—C22—C24	−174.8 (4)
Cd1—N5—C3—N6	178.53 (18)	C24—N1—C23—N2	1.0 (4)
C2—N6—C3—N5	−0.8 (3)	Cd2—N1—C23—N2	−172.7 (2)
C4—N6—C3—N5	−176.5 (3)	C22—N2—C23—N1	−1.3 (4)
C3—N6—C4—C5	99.4 (4)	C21—N2—C23—N1	174.7 (3)
C2—N6—C4—C5	−75.5 (4)	N2—C22—C24—N1	−0.4 (4)
C6—O4—C5—C4	177.2 (3)	C23—N1—C24—C22	−0.3 (4)
N6—C4—C5—O4	−66.5 (3)	Cd2—N1—C24—C22	172.7 (2)

Symmetry codes: (i) −*x*+1, −*y*, −*z*+1; (ii) −*x*, −*y*+1, −*z*+1.

### Hydrogen-bond geometry (Å, °)

**Table:**

*D*—H···*A*	*D*—H	H···*A*	*D*···*A*	*D*—H···*A*
O1W—H1WB···Cl2^iii^	0.85 (2)	2.33 (3)	3.165 (3)	164 (5)
O1W—H1WA···Cl1^iv^	0.88 (6)	2.42 (5)	3.198 (4)	147 (7)

Symmetry codes: (iii) *x*+1, *y*, *z*; (iv) −*x*+1, *y*+1/2, −*z*+1/2.
